# Weevil Knievels: attempting the leap for oceanic transport

**DOI:** 10.1093/conphys/coz014

**Published:** 2019-05-15

**Authors:** Christina Pasparakis

**Affiliations:** University of Miami, Rosenstiel School of Marine and Atmospheric Science, Miami, USA

Although dispersing across the oceans may seem like just a hop, skip and a splash away for fish or birds, terrestrial species that cannot fly face much greater challenges. They must find a sturdy and safe transport vessel if they are to move across the oceans to colonize new islands. Such vessels could include floating debris, wind or even other animals. But, much like the risky, daredevil stunts of the late Evel Knievel, the dangers of oceanic dispersal are profound.

So, is this ‘leap of faith’ worthwhile?

According to a team of researchers in Taiwan studying weevils, the answer is yes—at least for the 10% that actually survived their harrowing journeys.

Weevils are colorful beetles that are found mainly on the islands of Southeast Asia. Their eggs have a thick shell that is relatively impermeable, and both eggs and larvae live and grow inside a host plant. Perhaps not surprisingly, it was recently discovered that weevils could traverse oceans by hitching a ride on their host fruit. Indeed, all of these traits make them great candidates for Yeh and Tseng’s team in Taiwan to study oceanic transport.

To study this phenomenon, the team used field experiments but also laboratory simulations to test the survival rates of ocean-rafting weevils. The results of both field and laboratory experiments were consistent. Results indicated that about 10% of weevils attempting oceanic transport survived to tell the tale. So how do these little daredevils do it, and what factors determine their success?

To investigate this further, Yeh and Tseng’s team tested the salinity tolerances of weevils at different stages in development. Interestingly, weevil eggs and larvae were found to have a much better chance of survival in seawater than adults, suggesting that life stage is an important factor affecting the success of oceanic transport.

The integrity of host plants and their resilience to natural disturbances and changing environmental conditions is another important variable. The fruit of the fish poison tree, similar in many ways to coconuts, is a common host plant for weevils and were employed in these experiments due to a suite of characteristics thought to promote efficient oceanic dispersal. Such traits include a high salinity tolerance, a thick and waterproof outer shell and a middle layer containing air sacs that help facilitate floating.

Lastly, it was suggested that by exploiting fast moving currents (such as the Kuroshio current in the Pacific Ocean), weevils were able to reduce travel time and increase travel efficiency.

So the basis of success was, indeed, multifaceted.

From a conservation standpoint, studies similar to those conducted by Yeh and Tseng’s team are becoming increasingly important. In a time of such rapid and unpredictable climate change, the potential for dispersal and colonization of new habitats may determine a species’ ability to maintain sustainable populations and ultimately prevent extinction. But for now, our flightless and fearless friends seem to be floating along just fine.

**Cite as:** Yeh HY, Tseng HY, Lin CP, Liao CP, Hsu JY, Huang WS (2018) Rafting on floating fruit is effective for oceanic dispersal of flightless weevils. *J Exp Biol.* 221(24). doi:10.1242/jeb.190488.

Illustration by Erin Walsh; Email: ewalsh.sci@gmail.com



